# Supplementary material for: How inhibitory and excitatory inputs gate output of the inferior olive
Source: eLife. 2023 Aug 1;12:e83239. doi: 10.7554/eLife.83239 (PMC10393294; doi:10.7554/eLife.83239)
Supplement: Supplementary file 1. — (A) Kinetic properties of cerebellar nuclei (CN) and mesodiencephalic junction (MDJ) synaptic responses using different stimulation approaches. Latency and half-width of hyperpolarization (Vhyp) and rebound (Vreb) synaptic components of CN synaptic responses evoked in mice transduced with ChrimsonR-tdTomato in the CN and GAD2Cre/Chr2-H134R-EYFP transgenic mice line, and latency and half-width of depolarization (Vdep), hyperpolarization (Vhyp), and rebound (Vreb) synaptic components of MDJ synaptic response evoked in mice transduced with Chronos in the MDJ and electrical stimulation. (B) Phase response curve (PRC) parameters of different stimulation paradigms. Slope, Y-intercept, and R2 of PRCs generated by dual (CN and MDJ afferents stimulation) stimulation at different time intervals (all of them evoking subthreshold synaptic responses except for ‘+150 ms spike’ which evokes suprathreshold synaptic responses), CN afferent stimulation evoking subthreshold and suprathreshold synaptic responses (‘IPSP’ and ‘CN spike’, respectively), and MDJ afferent stimulation evoking suprathreshold synaptic responses (‘MDJ spike’). (C) Statistics of PRC slopes of different stimulation paradigms. Comparison of PRC slopes between +150 ms and the other time intervals (all of them evoking subthreshold synaptic responses except for ‘+150 ms spike’ which evokes suprathreshold synaptic responses), CN afferent stimulation evoking subthreshold and suprathreshold synaptic responses (‘IPSP’ and ‘CN spike’, respectively) and MDJ afferent stimulation evoking suprathreshold synaptic responses (‘MDJ spike’), using one-way ANOVA test followed by post hoc uncorrected Fisher’s LSD multiple comparison test. (D) Statistics of PRC Y-intercept. Comparison of PRC Y-intercepts between +150 ms and the other time intervals (all of them evoking subthreshold synaptic responses except for ‘+150 ms spike’ which evokes suprathreshold synaptic responses), CN afferent stimulation evoking subthreshold and suprathres [file elife-83239-supp1.docx]

**Supplemental Tables**

| **Source of the synaptic response** | **Type of stimulation** | **Latency of synaptic response (ms) (mean ± SD)** | **Half width V dep. (mean ± SD)** | **Half width V hyp. (mean ± SD)** | **Half width V reb. (mean ± SD)** |
| --- | --- | --- | --- | --- | --- |
| **CN** | **ChrimsonR** | **14.35 ± 6.74** | **--------** | **73.59 ± 14.97** | **79.74 ± 33.09** |
|  | **GAD2Cre/H134R** | **29.33 ± 10.08** | **--------** | **77.89 ± 7.49** | **82.98 ± 17.79** |
| **MDJ** | **Electrical** | **0.57 ± 0.23** | **12.06 ± 3.06** | **78.93 ± 11.09** | **79.16 ± 14.98** |
|  | **Chronos** | **2.26 ± 0.42** | **11.86 ± 5.03** | **80.90 ± 19.39** | **86.13 ± 12.96** |

**Supplementary file 1A. Kinetic properties of CN and MDJ synaptic responses using different stimulation approaches.** Latency and half width of hyperpolarization (V_hyp._) and rebound (V_reb._) synaptic components of CN synaptic responses evoked in mice transduced with ChrimsonR-tdTomato in the CN and GAD2Cre/Chr2-H134R-EYFP transgenic mice line, and latency and half width of depolarization (V_dep._), hyperpolarization (V_hyp._), and rebound (V_reb._) synaptic components of MDJ synaptic response evoked in mice transduced with Chronos in the MDJ and electrical stimulation.

| \| **Stimulation** \| **Slope ± SD** \| **Intercept ± SD** \| **r^2^** \| **Number of cells** \| \| --- \| --- \| --- \| --- \| --- \| \| **-100 ms** \| **-0.49 ± 0.03** \| **-0.16 ± 0.02** \| **0.69** \| **6** \| \| **-50 ms** \| **-0.41 ± 0.05** \| **-0.11 ± 0.02** \| **0.44** \| **6** \| \| **0 ms** \| **-0.56 ± 0.02** \| **0.08 ± 0.01** \| **0.88** \| **5** \| \| **+30 ms** \| **-0.53 ± 0.03** \| **0.02 ± 0.01** \| **0.78** \| **4** \| \| **+50 ms** \| **-0.51 ± 0.02** \| **0.02 ± 0.01** \| **0.74** \| **6** \| \| **+70 ms** \| **-0.45 ± 0.05** \| **0.05 ± 0.02** \| **0.5** \| **5** \| \| **+100 ms** \| **-0.48 ± 0.03** \| **0.09 ± 0.01** \| **0.71** \| **5** \| \| **+150 ms** \| **-0.69 ± 0.05** \| **0.35 ± 0.03** \| **0.61** \| **7** \| \| **+200 ms** \| **-0.51 ± 0.03** \| **0.08 ± 0.01** \| **0.74** \| **4** \| \| **IPSP** \| **-0.52 ± 0.01** \| **0.07 ± 0.01** \| **0.86** \| **5** \| \| **MDJ Spike** \| **-0.50 ± 0.02** \| **0.09 ± 0.01** \| **0.82** \| **5** \| \| **CN spike** \| **-0.54 ± 0.04** \| **0.15 ± 0.02** \| **0.78** \| **8** \| \| **+150 ms Spike** \| **-0.66 ± 0.04** \| **0.33 ± 0.02** \| **0.72** \| **7** \| |  |  |  |  |
| --- | --- | --- | --- | --- | --- | --- | --- | --- | --- | --- | --- | --- | --- | --- | --- | --- | --- | --- | --- | --- | --- | --- | --- | --- | --- | --- | --- | --- | --- | --- | --- | --- | --- | --- | --- | --- | --- | --- | --- | --- | --- | --- | --- | --- | --- | --- | --- | --- | --- | --- | --- | --- | --- | --- | --- | --- | --- | --- | --- | --- | --- | --- | --- | --- | --- | --- | --- | --- | --- | --- | --- | --- | --- | --- |
|  |  |  |  |  |
|  |  |  |  |  |
| **Supplementary file 1B**. **PRC parameters of different stimulation paradigms.** Slope, Y-intercept, and R^2^ of PRCs generated by dual (CN and MDJ afferents stimulation) stimulation at different time intervals (all of them evoking subthreshold synaptic responses except for ‘+150 ms spike’ which evokes suprathreshold synaptic responses), CN afferent stimulation evoking subthreshold and suprathreshold synaptic responses (‘IPSP’ and ‘CN spike’, respectively), and MDJ afferent stimulation evoking suprathreshold synaptic responses (‘MDJ spike’). |  |  |  |  |
|  |  |  |  |  |
|  |  |  |  |  |
|  |  |  |  |  |
|  |  |  |  |  |
|  |  |  |  |  |
|  |  |  |  |  |
|  |  |  |  |  |
|  |  |  |  |  |
|  |  |  |  |  |
|  |  |  |  |  |

| **Uncorrected Fisher's LSD** | **Mean Diff.** | **95.00% CI of diff.** | **P- value** |
| --- | --- | --- | --- |
| **+ 150 ms vs. -100 ms** | **-0.20** | **-0.31 to -0.10** | **0.0001** |
| **+ 150 ms vs. -50 ms** | **-0.28** | **-0.39 to -0.18** | **<0.0001** |
| **+ 150 ms vs. 0 ms** | **-0.13** | **-0.24 to -0.022** | **0.018** |
| **+ 150 ms vs. +30 ms** | **-0.17** | **-0.27 to -0.061** | **0.002** |
| **+ 150 ms vs. +50 ms** | **-0.18** | **-0.28 to -0.089** | **0.0001** |
| **+ 150 ms vs. +70 ms** | **-0.24** | **-0.34 to -0.14** | **<0.0001** |
| **+ 150 ms vs. +100 ms** | **-0.22** | **-0.32 to -0.11** | **<0.0001** |
| **+ 150 ms vs. +200 ms** | **-0.19** | **-0.29 to -0.082** | **0.0005** |
| **+ 150 ms vs. IPSP** | **-0.17** | **-0.26 to -0.078** | **0.0003** |
| **+ 150 ms vs. MDJ spike** | **-0.19** | **-0.29 to -0.096** | **<0.0001** |
| **+ 150 ms vs. CN spike** | **-0.15** | **-0.28 to -0.021** | **0.023** |
| **+ 150 ms vs. +150 ms spike** | **-0.029** | **-0.14 to 0.077** | **0.59** |

**Supplementary file 1C. Statistics of PRC slopes of different stimulation paradigms.** Comparison of PRC slopes between +150 ms and the other time intervals (all of them evoking subthreshold synaptic responses except for ‘+150 ms spike’ which evokes suprathreshold synaptic responses), CN afferent stimulation evoking subthreshold and suprathreshold synaptic responses (‘IPSP’ and ‘CN spike’, respectively) and MDJ afferent stimulation evoking suprathreshold synaptic responses (‘MDJ spike’), using one-way ANOVA test followed by post-hoc uncorrected Fisher’s LSD multiple comparison test.

| **Uncorrected Fisher's LSD** | **Mean Diff.** | **95.00% CI of diff.** | **P-value** |
| --- | --- | --- | --- |
| **+ 150 ms vs. -100 ms** | **0.52** | **0.46 to 0.58** | **<0.0001** |
| **+ 150 ms vs. -50 ms** | **0.46** | **0.41 to 0.52** | **<0.0001** |
| **+ 150 ms vs. 0 ms** | **0.27** | **0.21 to 0.33** | **<0.0001** |
| **+ 150 ms vs. +30 ms** | **0.33** | **0.27 to 0.39** | **<0.0001** |
| **+ 150 ms vs. +50 ms** | **0.33** | **0.28 to 0.38** | **<0.0001** |
| **+ 150 ms vs. +70 ms** | **0.30** | **0.24 to 0.36** | **<0.0001** |
| **+ 150 ms vs. +100 ms** | **0.26** | **0.20 to 0.32** | **<0.0001** |
| **+ 150 ms vs. +200 ms** | **0.26** | **0.21 to 0.32** | **<0.0001** |
| **+ 150 ms vs. IPSP** | **0.28** | **0.23 to 0.33** | **<0.0001** |
| **+ 150 ms vs. MDJ spike** | **0.26** | **0.20 to 0.31** | **<0.0001** |
| **+ 150 ms vs. CN spike** | **0.19** | **0.12 to 0.27** | **<0.0001** |
| **+ 150 ms vs. +150 ms spike** | **0.023** | **-0.037 to 0.083** | **0.45** |

**Supplementary file 1D. Statistics of PRC Y-intercept.** Comparison of PRC Y-intercepts between +150 ms and the other time intervals (all of them evoking subthreshold synaptic responses except for ‘+150 ms spike’ which evokes suprathreshold synaptic responses), CN afferent stimulation evoking subthreshold and suprathreshold synaptic responses (‘IPSP’ and ‘CN spike’, respectively) and MDJ afferent stimulation evoking suprathreshold synaptic responses (‘MDJ spike’) using one-way ANOVA test followed by post-hoc uncorrected Fisher’s LSD multiple comparison test.

| **Stimulation** | **Intertrial phase jitter (Standard deviation of phase lags) pre- stimulus (ms)** | **Intertrial phase jitter (Standard deviation of phase lags) post-stimulus (ms)** | **Intertrial phase jitter change (Standard deviation of phase lags post stimulus – Standard deviation of phase lags pre- stimulus)** | **Number of cells** |
| --- | --- | --- | --- | --- |
| **-100 ms** | **60.84** | **13.47** | **-47.37** | **10** |
| **-50 ms** | **58.39** | **19.86** | **-38.53** | **13** |
| **0 ms** | **56.87** | **14.84** | **-42.03** | **14** |
| **+30 ms** | **62.36** | **16.61** | **-45.74** | **17** |
| **+50 ms** | **64.84** | **36.48** | **-28.35** | **35** |
| **+70 ms** | **73.35** | **28** | **-45.34** | **17** |
| **+100 ms** | **57.51** | **18.13** | **-39.38** | **16** |
| **+150 ms** | **49.17** | **14.88** | **-34.98** | **10** |
| **+200 ms** | **52.61** | **10.62** | **-41.99** | **10** |
| **IPSP** | **59.51** | **20.35** | **-39.16** | **15** |
| **EPSP** | **56.47** | **44.97** | **-11.50** | **15** |
| **MDJ Spike** | **51.92** | **36.05** | **-9.29** | **8** |
| **+150 ms Spike** | **51.47** | **3.21** | **-48.26** | **7** |
| **CN spike** | **61.05** | **38.1** | **-22.95** | **7** |

**Supplementary file 1E. Intertrial phase jitter at different stimulation paradigms.** Intertrial phase jitter pre- and post-stimulus (Standard deviation of phase lags pre and post- stimulus, respectively) and intertrial phase jitter change (Standard deviation of phase lags post stimulus – Standard deviation of phase lags pre-stimulus) at different time intervals of stimulation (all of them evoking subthreshold synaptic responses except for ‘+150 ms spike’ which evokes suprathreshold synaptic responses), CN afferent stimulation evoking subthreshold and suprathreshold synaptic responses (‘IPSP’ and ‘CN spike’, respectively) and MDJ afferent stimulation evoking subthreshold and suprathreshold synaptic responses (‘EPSP’ and ‘MDJ spike’, respectively).

| **Time interval** | **Uncorrected Dunn's test** | **Rank sum diff.** | **P-value** |
| --- | --- | --- | --- |
| **-100 ms** | **Δ Pre stim STO average vs. Post stim STO 1st peak** | **-276** | **<0.0001** |
| **-50 ms** | **ΔPre stim STO average vs. Post stim STO 1st peak** | **-346** | **<0.0001** |
| **0 ms** | **ΔPre stim STO average vs. Post stim STO 1st peak** | **-396** | **<0.0001** |
| **+30 ms** | **ΔPre stim STO average vs. Post stim STO 1st peak** | **-402** | **<0.0001** |
| **+ 50ms** | **ΔPre stim STO average vs. Post stim STO 1st peak** | **-452** | **<0.0001** |
| **+70 ms** | **ΔPre stim STO average vs. Post stim STO 1st peak** | **-53** | **0.07** |
| **+100 ms** | **ΔPre stim STO average vs. Post stim STO 1st peak** | **-440** | **<0.0001** |
| **+150 ms** | **ΔPre stim STO average vs. Post stim STO 1st peak** | **-307** | **<0.0001** |
| **+200 ms** | **ΔPre stim STO average vs. Post stim STO 1st peak** | **-367** | **<0.0001** |

**Supplementary file 1F. Statistics of amplitude of first cycle following stimulation at different time intervals.** Comparison between STO amplitude change of the first cycle (rebound component) following stimulation at different time intervals (all of them evoking subthreshold synaptic responses) and STO amplitude previous to stimulation using Friedman test followed by post-hoc uncorrected Dunn’s multiple comparison test.

| **Stimulation** | **STO amplitude change of 1^st^ cycle post-stim ± SD (mV)** | **Number of cells** |
| --- | --- | --- |
| **-100 ms** | **6.18 ± 4.98** | **10** |
| **-50 ms** | **5.53 ± 5.03** | **13** |
| **0 ms** | **4.80 ± 4.32** | **14** |
| **+30 ms** | **3.66 ± 2.98** | **16** |
| **+50 ms** | **1.83 ± 3.42** | **34** |
| **+70 ms** | **0.48 ± 2.59** | **17** |
| **+100 ms** | **6.48 ± 3.47** | **16** |
| **+150 ms** | **7.25 ± 3.45** | **10** |
| **+200 ms** | **4.08 ± 2.05** | **10** |
| **IPSP** | **4.82 ± 3.70** | **23** |
| **EPSP** | **1.52 ± 3.04** | **26** |
| **MDJ Spike** | **6.75 ± 4.26** | **10** |
| **CN spike** | **4.04** **± 3.19** | **6** |
| **+150 ms spike** | **8.67 ± 5.03** | **6** |

**Supplementary file 1G. STO amplitude change of first cycle following stimulation at different stimulation paradigms.** STO amplitude change of the first cycle (rebound component) following dual stimulation at different time intervals (all of them evoking subthreshold synaptic responses except for “+150 ms spike” which evokes suprathreshold synaptic responses),CN afferent stimulation evoking subthreshold and suprathreshold synaptic responses (‘IPSP’ and ‘CN spike’, respectively), and MDJ afferent stimulation evoking subthreshold and suprathreshold synaptic responses (‘EPSP’ and ‘MDJ spike’, respectively).

| **Uncorrected Dunn's test** | **Mean rank diff.** | **P-value** |
| --- | --- | --- |
| **+150 ms vs. -100 ms** | **227.0** | **0.008** |
| **+150 ms vs. -50 ms** | **277.1** | **0.0005** |
| **+150 ms vs. 0 ms** | **359.1** | **<0.0001** |
| **+150 ms vs. +30 ms** | **522.2** | **<0.0001** |
| **+150 ms vs. +50 ms** | **787.1** | **<0.0001** |
| **+150 ms vs. +70 ms** | **1042** | **<0.0001** |
| **+150 ms vs. +100 ms** | **88.41** | **0.25** |
| **+150 ms vs. +200 ms** | **420.1** | **<0.0001** |
| **+150 ms vs. EPSP** | **867.1** | **<0.0001** |
| **+150 ms vs. IPSP** | **367.4** | **<0.0001** |
| **+150 ms vs. MDJ spike** | **110.1** | **0.19** |
| **+150 ms vs. CN spike** | **456.8** | **<0.0001** |
| **+150 ms vs. +150 ms spike** | **-37.74** | **0.68** |

**Supplementary file 1H . Statistics of the comparison between first cycle post-stimulus change evoked by +150 ms and other stimulation paradigms.** Comparison of first cycle post-stimulus change between the time interval of +150 ms and the other time intervals (all of them evoking subthreshold synaptic responses except for “+150 ms spike” which evokes suprathreshold synaptic responses), CN afferent stimulation evoking subthreshold and suprathreshold synaptic responses (‘IPSP’ and ‘CN spike’, respectively), and MDJ afferent stimulation evoking subthreshold and suprathreshold synaptic responses (‘EPSP’ and ‘MDJ spike’, respectively), using Kruskal-Wallis test followed by post-hoc uncorrected Dunn’s multiple comparison test.

| **Stimulation** | **P_spike_ ± SD** | **Number of cells** |
| --- | --- | --- |
| **-100 ms** | **0.16 ± 0.22** | **9** |
| **-50 ms** | **0.17 ± 0.26** | **9** |
| **0 ms** | **0.23 ± 0.39** | **7** |
| **+30 ms** | **0.05 ± 0.09** | **7** |
| **+50 ms** | **0.0 ± 0.0** | **9** |
| **+70 ms** | **0.0 ± 0.0** | **6** |
| **+100 ms** | **0.08 ± 0.21** | **8** |
| **+150 ms** | **0.57 ± 0.30** | **9** |
| **+200 ms** | **0.26 ± 0.22** | **7** |
| **IPSP** | **0.01 ± 0.03** | **7** |
| **EPSP** | **0.22 ± 0.28** | **7** |

**Supplementary file 1I**. **Spike probability at different stimulation paradigms.** Spike probability (P_spike_) following dual stimulation at different time intervals and isolated CN and MDJ afferents stimulation (‘IPSP’ and ‘MDJ’, respectively).

| **Uncorrected Fisher's LSD** | **Mean Diff.** | **95.00% CI of diff.** | **P-value** |
| --- | --- | --- | --- |
| **+150 ms vs. -100 ms** | **0.41** | **0.21 to 0.62** | **0.0002** |
| **+150 ms vs. -50 ms** | **0.4** | **0.19 to 0.61** | **0.0002** |
| **+150 ms vs. 0ms** | **0.34** | **0.13 to 0.54** | **0.0015** |
| **+150 ms vs. +30 ms** | **0.52** | **0.30 to 0.74** | **<0.0001** |
| **+150 ms vs. +50 ms** | **0.58** | **0.37 to 0.78** | **<0.0001** |
| **+150 ms vs. +70 ms** | **0.58** | **0.35 to 0.81** | **<0.0001** |
| **+150 ms vs. +100 ms** | **0.49** | **0.28 to 0.70** | **<0.0001** |
| **+150 ms vs. +200 ms** | **0.31** | **0.094 to 0.53** | **0.0057** |
| **+150 ms vs. EPSPs only** | **0.36** | **0.14 to 0.58** | **0.0018** |
| **+150 ms vs. IPSP only** | **0.56** | **0.34 to 0.78** | **<0.0001** |

**Supplemental file 1J. Statistics of the comparison between spike probability evoked by +150 ms and other stimulation paradigms.** Comparison of spike probability (P_spike_) of time interval of +150 ms and all the other time intervals and CN and MDJ isolated afferents stimulation ( ‘IPSP’ and ‘EPSP’, respectively) using one-way anova test followed by post-hoc uncorrected Fisher’s LSD multiple comparison test.
