# Supplementary material for: How inhibitory and excitatory inputs gate output of the inferior olive
Source: eLife. 2023 Aug 1;12:e83239. doi: 10.7554/eLife.83239 (PMC10393294; doi:10.7554/eLife.83239)
Supplement: MDAR checklist [file elife-83239-mdarchecklist1.pdf]

Westbrook Centre
Milton Road
Cambridge CB4 1YG, UK

P 01223 855340
W elifesciences.org
T @elife

Materials Design Analysis Reporting (MDAR) Checklist for Authors

The MDAR framework establishes a minimum set of requirements in transparent reporting mainly applicable to studies in the life sciences.

eLife asks authors to ***provide detailed information within their article*** *to facilitate the interpretation and replication of their work. Authors can also upload supporting materials to comply with relevant reporting guidelines for health-related research (see EQUATOR Network), life science research (see the BioSharing Information Resource), or animal research (see the ARRIVE Guidelines and the STRANGE Framework; for details, see eLife's Journal Policies). Where applicable, authors should refer to any relevant reporting standards materials in this form.*

For all that apply, please note ***where in the article*** *the information is provided. Please note that we also collect information about data availability and ethics in the submission form.*

Materials:

Newly created materials	*Indicate where provided: section/figure legend*	*N/A*
The manuscript includes a dedicated "materials availability statement" providing transparent disclosure about availability of newly created materials including details on how materials can be accessed and describing any restrictions on access.		*N/A*

Antibodies	*Indicate where provided: section/figure legend*	*N/A*
For commercial reagents, provide supplier name, catalogue number and RRID, if available.		*N/A*

DNA and RNA sequences	*Indicate where provided: section/figure legend*	*N/A*
Short novel DNA or RNA including primers, probes: Sequences should be included or deposited in a public repository.		*N/A*

Cell materials	*Indicate where provided: section/figure legend*	*N/A*
Cell lines: Provide species information, strain. Provide accession number in repository OR supplier name, catalog number, clone number, OR RRID.		*N/A*
Primary cultures: Provide species, strain, sex of origin, genetic modification status.		*N/A*

Experimental animals	*Indicate where provided: section/figure legend*	*N/A*
Laboratory animals or Model organisms: Provide species, strain, sex, age, genetic modification status. Provide accession number in repository OR supplier name, catalog number, clone number, OR RRID.	C57/BL6 mice (Figures 1, 2,3,4,5,6) and GAD2-Cre x Ai32 transgenic mice (Figures: 2,4,5 and 6). Either sex was used for both experiment models and their age for experimental use ranged from 1 to 3 months old.	
Animal observed in or captured from the field: Provide species, sex, and age where possible.		*N/A*

Plants and microbes	*Indicate where provided: section/figure legend*	*N/A*
Plants: provide species and strain, ecotype and cultivar where relevant, unique accession number if available, and source (including location for collected wild specimens).		*N/A*
Microbes: provide species and strain, unique accession number if available, and source.		*N/A*

Human research participants	*Indicate where provided: section/figure legend) or state if these demographics were not collected*	*N/A*
If collected and within the bounds of privacy constraints report on age, sex, gender and ethnicity for all study participants.		*N/A*

Design:

Study protocol	*Indicate where provided: section/figure legend*	*N/A*
If the study protocol has been pre-registered, provide DOI. For clinical trials, provide the trial registration number OR cite DOI.		*N/A*

Laboratory protocol	*Indicate where provided: section/figure legend*	*N/A*

Provide DOI OR other citation details if detailed step-by-step protocols are available.	*All protocols are stated in the methods section of the article*	

*Experimental study design (statistics details) **		
For in vivo studies: State whether and how the following have been done	***Indicate where provided: section/figure legend. If it could have been done, but was not, write "not done"***	***N/A***
Sample size determination		*N/A*
Randomisation		*N/A*
Blinding		*N/A*
Inclusion/exclusion criteria		*N/A*

Sample definition and in-laboratory replication	***Indicate where provided: section/figure legend***	***N/A***
State number of times the experiment was replicated in the laboratory.		*N/A*
Define whether data describe technical or biological replicates.		*N/A*

Ethics	***Indicate where provided: section/submission form***	***N/A***
Studies involving human participants: State details of authority granting ethics approval (IRB or equivalent committee(s), provide reference number for approval.		
Studies involving experimental animals: State details of authority granting ethics approval (IRB or equivalent committee(s), provide reference number for approval.	*All performed experiments were licensed by the Dutch Competent Authority and approved by the local Animal Welfare Body, following the European guidelines for the care and use of laboratory animals Directive 2010/63/EU.*	
Studies involving specimen and field samples: State if relevant permits obtained, provide details of authority approving study; if none were required, explain why.		

Dual Use Research of Concern (DURC)	***Indicate where provided: section/submission form***	*N/A*
If study is subject to dual use research of concern regulations, state the authority granting approval and reference number for the regulatory approval.		*N/A*

Analysis:

Attrition	***Indicate where provided: section/figure legend***	***N/A***
Describe whether exclusion criteria were pre-established. Report if sample or data points were omitted from analysis. If yes, report if this was due to attrition or intentional exclusion and provide justification.	***Figure 1 Panels D and E.*** *two oscillating cells were excluded from analysis because their oscillation amplitude were lower than 1 mV because that very low oscillatory activity could be due to noise and not from a biological signal.*<br><br>***Section "cell physiological properties":*** *3 cells were excluded from the estimation of biocytin labeled coupled cells because they were obtained from coronal slices and not from sagittal slices (all of the cells used for the paper were obtained from sagittal slices)*<br><br>***Figure 2 and 3 Panel E:*** *Only trials in which oscillations were absent previous to stimulation were selected because the idea of those experiments was to assess the impact of synaptic inputs on induced oscillations, which requires a non-oscillating state previous to stimulation.*<br><br>***Figure 2 and 3: Panels G and H, Figure 5: PRCs curves and Phase Lags plots:*** *Traces excluded were those in which excitation or inhibition elicited spikes, STOs were irregular, transiently obliterated, or spikes were spontaneously triggered either before or after the subthreshold synaptic response evoked by stimulation. Those traces were excluded because only subthreshold synaptic responses effect on regular oscillating neurons were assessed in those experiments, otherwise PRCs could be altered by spike's phase resetting effect and*	

	irregular oscillations.<br><br>***Figure 6, PRCs curves and Phase Lags:*** *Traces excluded from analysis were those in which STOs were irregular, transiently obliterated, or spikes were spontaneously triggered either before or after the suprathhreshold response elicited by the synaptic stimulation. Those trials were excluded because only suprathrehold synaptic responses effect on regular oscillating neurons were assessed in those experiments, otherwise PRCs could be altered by spike's phase resetting effect (either before or after the suprathreshold response) and irregular oscillations.*<br><br>***Figure 2 and 3 Panel, Figure 5 panel F (third figure from top to bottom), and Figure 6 (fourth column from left to right, middle panel):*** *Due to a possible big overrepresentation of data points derived from some cells, 10 trials were randomly selected from cells that exceed that number of trials. For this purpose a Matlab code was used (also available called 'Random Sampling').Indexes from data matrices are available in order to track which trials were selected for each cell. Furthermore, trials in which oscillations were absent were not included since impact of synaptic input on STOs was assessed.*<br><br>***Section: MDJ input and subthreshold activity of IO neurons:*** *Regarding the estimation of STOs duration induced by EPSPs and IPSPs, trials excluded were those in which STOs duration exceeds either the swipe length of the recording or the interstimulus interval because in those cases the measurement of the STO duration was not accurate.*	

Statistics	*Indicate where provided: section/figure legend*	*N/A*
Describe statistical tests used and justify choice of tests.	***Figures: 1,2,3,5 and 6***<br><br>*All statistical tests used for each of the experiments of the paper are clearly stated in the main text.*<br><br>*All statistical analysis were performed in GraphPad Prism 8.3.0. In order to test whether the data was normally distributed, D'Agostino-Pearson omnibus normality test was performed. For comparisons of two groups showing normally distributed data, unpaired or paired t-test was performed, whereas for comparison of two groups showing non-normally distributed data, Mann-Whitney U test or Wilcoxon matched-paired rank test was performed depending if data was unpaired or paired, respectively. When multiple groups normally distributed were compared, One-Way ANOVA test followed by post-hoc uncorrected Fisher's LSD multiple comparison test was used. When multiple groups were not normally distributed and data was not paired, Kruskal-Wallis test followed by uncorrected post-hoc Dunn's test was used, whereas in case that multiple multiple groups were not normally distributed and data was paired, Friedman test followed by post-hoc uncorrected Dunn's test was used. To determine whether the slope and intercept of two linear regressions were significantly different ANCOVA test was used, whereas for multiple comparisons one-way ANOVA test followed by post-hoc uncorrected Fisher's LSD multiple comparison test was used.*<br>*To determine the impact of stimulation on intertrial phase jitter, F test was used to compare the standard deviation pre and post-stimulus ($SD_{pre-stim}$ and $SD_{post-stim}$, respectively). Moreover, to assess and compare the impact of different types of stimulation on intertrial phase jitter, F test was used to compare the values resulted from the subtraction of $SD_{post-stim}$ and $SD_{pre-stim}$, called $\Delta SD_{post-pre}$, across different*	

	experimental conditions. For all the aforementioned tests, P values below 0.05 were considered statistically significant.	

Data availability	*Indicate where provided: section/submission form*	*N/A*
For newly created and reused datasets, the manuscript includes a data availability statement that provides details for access (or notes restrictions on access).		
When newly created datasets are publicly available, provide accession number in repository OR DOI and licensing details where available.	*All data used for the figures and codes for the analysis are stored in the following DOI: 10.5061/dryad.pg4f4qrtd*	
If reused data is publicly available provide accession number in repository OR DOI, OR URL, OR citation.		

Code availability	*Indicate where provided: section/figure legend*	*N/A*
For any computer code/software/mathematical algorithms essential for replicating the main findings of the study, whether newly generated or re-used, the manuscript includes a data availability statement that provides details for access or notes restrictions.		
Where newly generated code is publicly available, provide accession number in repository, OR DOI OR URL and licensing details where available. State any restrictions on code availability or accessibility.	*Folder called "Figures_4, 7 and 8_Codes for simulation (OliveTree-master)" contains Contains all codes to recreate datasets and figures 4, 7 y 8. The scripts producing all figures and data are: Loyola_PRC.m and Exc_Inh_Reconstruction.m*<br><br>*URL: https://www.dropbox.com/s/3i8ndqyvyrunxxp/Figures_4%2C%207%20and%208_Codes%20for%20simulation%20%28OliveTree-master%29.zip?dl=0*<br><br>*Folder called "Codes Matlab Figure 1,2,3,5,6" contains the codes used*	

	for analysis of the figures 1,2,3, 5 and 6. Their main codes are Plot ABF: Matlab data structures were generated PrePostSTO: Calculate the amplitude change post-stimulus with respect to pre-stimulus baseline. It needs the matlab structures in order to do that. Hist_Overlap: It generates phase lags histograms.<br><br>*URL: https://www.dropbox.com/s/9055n0s5enin9cr/Codes%20Matlab%20Figure%201%2C2%2C3%2C5%2C6.rar?dl=0*	
If reused code is publicly available provide accession number in repository OR DOI OR URL, OR citation.		

Reporting:

The MDAR framework recommends adoption of discipline-specific guidelines, established and endorsed through community initiatives.

Adherence to community standards	*Indicate where provided: section/figure legend*	*N/A*
State if relevant guidelines (e.g., ICMJE, MIBBI, ARRIVE, STRANGE) have been followed, and whether a checklist (e.g., CONSORT, PRISMA, ARRIVE) is provided with the manuscript.		*N/A*

---

* We provide the following guidance regarding transparent reporting and statistics; we also refer authors to Ten common statistical mistakes to watch out for when writing or reviewing a manuscript.

Sample-size estimation

- You should state whether an appropriate sample size was computed when the study was being designed
- You should state the statistical method of sample size computation and any required assumptions
- If no explicit power analysis was used, you should describe how you decided what sample (replicate) size (number) to use

Replicates

- You should report how often each experiment was performed
- You should include a definition of biological versus technical replication
- The data obtained should be provided and sufficient information should be provided to indicate the number of independent biological and/or technical replicates
- If you encountered any outliers, you should describe how these were handled
- Criteria for exclusion/inclusion of data should be clearly stated

- High-throughput sequence data should be uploaded before submission, with a private link for reviewers provided (these are available from both GEO and ArrayExpress)

Statistical reporting

- Statistical analysis methods should be described and justified
- Raw data should be presented in figures whenever informative to do so (typically when N per group is less than 10)
- For each experiment, you should identify the statistical tests used, exact values of N, definitions of center, methods of multiple test correction, and dispersion and precision measures (e.g., mean, median, SD, SEM, confidence intervals; and, for the major substantive results, a measure of effect size (e.g., Pearson's r, Cohen's d)
- Report exact p-values wherever possible alongside the summary statistics and 95% confidence intervals. These should be reported for all key questions and not only when the p-value is less than 0.05.

Group allocation

- Indicate how samples were allocated into experimental groups (in the case of clinical studies, please specify allocation to treatment method); if randomization was used, please also state if restricted randomization was applied
- Indicate if masking was used during group allocation, data collection and/or data analysis
